# Travel-associated gonorrhoea in four Nordic countries, 2008 to 2013

**DOI:** 10.2807/1560-7917.ES.2017.22.20.30537

**Published:** 2017-05-18

**Authors:** Julien Beauté, Susan Cowan, Eija Hiltunen-Back, Hilde Kløvstad, Inga Velicko, Gianfranco Spiteri

**Affiliations:** 1 European Centre for Disease Prevention and Control (ECDC), Stockholm, Sweden; 2 Statens Serum Institut (SSI), Copenhagen, Denmark; 3 National Institute for Health and Welfare (THL), Helsinki, Finland; 4 Norwegian Institute of Public Health (FHI), Oslo, Norway; 5 Public Health Agency of Sweden, Stockholm, Sweden

**Keywords:** Sexually Transmitted Infections, Gonorrhoea, Travel, Scandinavian and Nordic Countries, Surveillance

## Abstract

Travel may be associated with a higher risk of gonorrhoea and infection by antibiotic-resistant strains. The objective of this study was to estimate the risk for gonorrhoea among travellers from four Nordic European countries using surveillance data and to identify at-risk travellers to help target interventions. We retrieved gonorrhoea surveillance data from Denmark, Finland, Norway and Sweden and tourism denominator data from the Statistical Office of the European Union. A travel-associated case of gonorrhoea was defined as one for which the reported country of infection differed from the reporting country. During 2008−2013, the four countries reported 3,224 travel-associated gonorrhoea cases, of which 53% were among individuals below 35 years of age. The overall risk associated with travel abroad was 2.4 cases per million nights abroad. The highest risk was observed with travel to Asia (9.4). Cases more likely to be reported as travel-associated were: males, heterosexuals of both sexes, people older than 65 years, and foreign-born individuals. More effective interventions targeting young adults and other at-risk groups are needed. The use of travel-planning websites and social media should be explored further.

## Background

Gonorrhoea is a common sexually transmitted infection (STI). Although gonorrhoea can be asymptomatic, particularly in women and in pharyngeal and rectal infections, it is a major cause of urethritis in both men and women. In women, infection can lead to pelvic inflammatory disease, which may cause infertility [1].

The World Health Organization estimates that in 2012 there were over 78 million cases of gonorrhoea globally [2]. In 2013, 52,995 cases of gonorrhoea were reported in European Union (EU)/European Economic Area (EEA) countries (16.9 cases per 100,000 population) [3]. Of these, 2,701 (5.1%) were reported by Denmark, Finland, Norway and Sweden. Notification rate was close to the EU/EEA average in Denmark, Norway and Sweden (10–15 cases per 100,000 population) but lower in Finland (4.9). In these countries, most cases were reported among 25–34 year olds (960 cases; 36%) and among young adults aged 15–24 (908 cases; 34%). Men accounted for 75% of cases (2,012 cases) and 39% (1,050 cases) were reported among men who have sex with men (MSM). The number of reported cases has increased by 61% since 2009.

MSM are a key at-risk group for infection and accounted for 43% of gonorrhoea cases in the EU/EEA in 2013 [3]. The risk of transmission of gonorrhoea can be reduced by consistent and correct condom use during sex [4]. Apart from primary prevention and partner notification, effective treatment is the only option for control of gonorrhoea in the absence of an effective vaccine. European and national treatment guidelines are available [5]. An increasing number of *Neisseria gonorrhoeae* strains have been reported with reduced susceptibility to antimicrobials used for treatment [6].

The relation between travel and infectious diseases is of public health concern for two main reasons. First, travel can facilitate the international spread of diseases. Second, travel may be associated with an additional disease burden due to both different risk and exposure in the destination country compared with the country of origin. Such specific risk may justify targeted preventive measures such as vaccination, prophylaxis [7], or in the case of STIs, the provision of advice before departure and testing upon return.

The historical role of travellers in spreading STIs is well documented [8]. Travel is also a known risk factor for STIs for a number of reasons, including changes in sexual behaviour when travelling [8]. Thus, travel has been shown to remove social taboos and to increase the likelihood of casual sexual relationships [9]. Travel is also associated with low condom use and sex may be the main objective of the journey (sex tourism) [9]. Travellers might also have sex with populations with higher prevalence of STIs, such as sex workers, or they might be visiting countries with a high prevalence of STI and therefore increase their risk of infection [9]. In addition, the emergence of antibiotic-resistant strains of gonorrhoea has been linked to countries in south-east Asia and Japan, and therefore travellers might import such strains to Europe [10]. Similarly, the prevalence of strains resistant to antimicrobials might be higher in further countries/regions outside Europe and lead to importation of resistant strains [9].

Estimating the real risk of travel-associated STIs is challenging. Data, particularly from Europe, are limited, notably because of reporting biases, incompleteness of STI surveillance data [8] and difficulties in obtaining sound data on travel patterns [11]. Yet, with an increasing number of travellers, it is important to better document, prevent and control travel-associated STI. Indeed, over the past 20 years, global tourist departures have doubled to reach half a billion in 2013, and Europe accounts for half of them [12].

The objective of this study is to estimate the risk for gonorrhoea among travellers from four Nordic countries using surveillance and tourism denominator data and to identify at-risk travellers to help target interventions.

## Methods

### Gonorrhoea data

The epidemiological surveillance of gonorrhoea in Europe is carried out at the national level by EU/EEA countries’ public health institutes or similar bodies. At the European level, representatives from the countries form the European Network for STI Surveillance, which is coordinated by the European Centre for Disease Prevention and Control (ECDC). The network comprises all 28 EU countries, plus Iceland and Norway. Each year, all diagnosed cases meeting the EU case definition for gonorrhoea in the previous year are reported to The European Surveillance System (TESSy) database hosted by ECDC [13]. Cases are reported with a set of variables including age, sex, probable mode of transmission, country of birth, and probable country of infection. In addition, a separate and unlinked surveillance system, the European Gonococcal Antimicrobial Surveillance Programme (Euro-GASP), collects epidemiological and antimicrobial resistance data on a subset of isolates from sentinel laboratories in participating Member States. Euro-GASP surveillance data were not analysed due to the very low completeness of reported epidemiological data on the probable country of infection.

For the purpose of this analysis, we included epidemiological surveillance data from four Nordic countries (Denmark, Finland, Norway and Sweden) for the years 2008−2013. These countries (total population 26 million) have surveillance systems with comprehensive coverage of the population and have reported continuously during the study period (i.e. at least one travel-associated case each year). In addition, Nordic countries share a number of similarities in their outbound tourism patterns, with trips more evenly distributed across the year [14], which support a pooled analysis. A travel-associated case of gonorrhoea was defined as one for which the reported probable country of infection differed from the reporting country. A foreign-born case was defined as a case with a country of birth different from the reporting country.

### Tourism data

Tourism denominator data for 2008−2013 were obtained from the Statistical Office of the EU (Eurostat) [15]. We used the total number of nights spent by destination country. This included all tourism nights spent by EU/EEA residents aged 15 years or over, in a collective accommodation establishment or in private tourism accommodation for personal or professional purposes. In most countries, this information is collected through household surveys. Number of nights by sex and age was only available for Denmark. Since tourism nights were not available for all country-region combinations for all years, we calculated the mean of tourism nights for the available years over the study period. To obtain a denominator for the entire study period (6 years), the mean number of tourism nights was then multiplied by six.

### Analysis

Travel-associated cases of gonorrhoea were compared with non-travel-associated cases for main characteristics. Risk for travel-associated gonorrhoea was calculated by dividing the number of travel-associated cases by the number of nights spent in the destination country. For Denmark, risk was also calculated by sex and age group. For all countries, risk was additionally calculated by region of travel. We considered five main regions (Africa, the Americas, Asia, Europe and Oceania). We divided the Americas further into North America (including Greenland) and Central and South America (including Mexico). In Europe, we distinguished EU countries from other European countries (including Turkey).

Continuous variables were compared across strata by the Mann–Whitney U test. Categorical variables were compared using the chi-squared or Fisher exact tests.

To assess the association of different variables with travel-associated gonorrhoea, we performed a multivariable logistic regression using surveillance data and estimated adjusted odds ratios (OR) with their 95% confidence intervals (CI). We adjusted for sex, age, route of transmission, country of residence, origin and year.

## Results

During the 2008−2013 period, Denmark, Finland, Norway and Sweden reported 12,645 gonorrhoea cases, of which 11,407 (90.2%) had information on probable country of infection. (Table 1).

**Table 1:** Main characteristics of reported cases with gonorrhoea infection by probable travel status, Denmark, Finland, Norway and Sweden, 2008–2013 (n = 12,645)

	Total	Travel‑associated cases		Non‑travel‑associated cases	
**Characteristic**	**Number**	**Number**	**%**	**Number**	**%**
**Total**	**12,645**	**3,224**	**100**	**9,421**	**100**
Sex					
Male	9,635	2,755	85.5	6,880	73.0
Female	3,009	469	14.5	2,540	27.0
Unknown	1	0	NA	1	NA
Age group (years)					
< 15	21	2	0.1	19	0.2
15–24	4,438	780	24.2	3,658	38.8
25–34	4,142	940	29.2	3,202	34.0
35–44	2,293	730	22.6	1,563	16.6
45–54	1,181	488	15.1	693	7.4
55–64	430	202	6.3	228	2.4
≥ 65	139	82	2.5	57	0.6
Unknown	1	NA	NA	1	NA
Transmission					
Heterosexual	7,149	2196	76.8	4,953	58.4
MSM	4,143	652	22.8	3,491	41.2
Other	46	10	0.3	36	0.4
Unknown	1,307	366	NA	941	NA
Country of residence					
Denmark	3,445	365	11.3	3,080	32.7
Finland	1,558	531	16.5	1,027	10.9
Norway	2,299	790	24.5	1,509	16.0
Sweden	5,343	1,538	47.7	3,805	40.4
Origin					
Native	4,824	939	84.8	3,885	94.9
Foreign-born	376	168	15.2	208	5.1
Unknown	7,445	2,117	NA	5,328	NA
Year					
2008	1,632	460	14.3	1,172	12.4
2009	1,683	461	14.3	1,222	13.0
2010	1,991	568	17.6	1,423	15.1
2011	2,109	575	17.8	1,534	16.3
2012	2,526	571	17.7	1,955	20.8
2013	2,704	589	18.3	2,115	22.4

MSM: men who have sex with men; NA: not applicable.

Of the 12,645 reported cases, 3,224 (25.5%) were travel-associated. This proportion fluctuated between 27.3% and 28.5% from 2008 to 2011 and then dropped to 22.6% in 2012 and 21.8% in 2013. Over the period, the annual number of cases continuously increased from 1,632 in 2008 to 2,704 in 2013 (+ 65.7%). This increase was observed for both travel-associated and non-travel-associated cases, but the increase was more pronounced in non-travel-associated cases (+ 80.5% vs + 28.0%). Finland, Norway and Sweden had similar proportions of travel-associated cases (ca 30%) while only 10.6% of Danish cases were related to travel.

### Demographics

Of the 3,224 travel-associated cases, 2,755 (85.5%) were male, giving a male-to-female ratio of 5.9:1 (Table 1). Overall, the male-to-female ratio increased with age (1.8:1 below 25 years, 6.7:1 for the 25–34 years and > 15 above 35 years). Cases in males were more often travel-associated than cases in females (28.6% vs 15.6%, p < 0.01). The proportion of females reported with gonorrhoea infections increased over the study period from below 20% in 2008 to over 25% in 2011−2013. Similarly, the proportion of females among travel-associated cases increased from 10% in 2008 to 15–19% in 2011−2013. Travel-associated cases were older than non-travel-associated cases (median age at diagnosis 33 vs 27 years, respectively, p < 0.01). Median ages of both travel-associated and non-travel-associated cases were stable over the study period. The proportion of travel-associated cases increased with age from 17.5% below 25 years to 59.0% at 65 years and above. Of the 3,224 travel-associated cases, 1,722 (53.4%) were young adults below 35 years of age; among non-travel-associated cases, young adults accounted for 73.0% of cases. Information on country of origin was available in Denmark, Finland and Norway. Of the 5,200 cases with known country of origin, 376 (7.2%) were foreign-born. Foreign-born cases were more often travel-associated than native-born cases (15.2% vs 5.1%, p < 0.01).

### Transmission

Of the 2,858 (88.6%) travel-associated cases with known probable route of transmission, 2,196 (76.8%) were reported as heterosexual transmission, 652 (22.8%) were due to sex between men, and 10 (0.3%) were through other routes of transmission (Table 1). Of the 2,432 travel-associated cases in males with known transmission status, 652 (26.8%) were MSM, ranging from 19.4% (58/299) in Denmark to 31.7% (230/725) in Norway. The proportion of travel-associated cases was higher in heterosexually transmitted cases compared with MSM (30.7% vs 15.7%, p < 0.01) and this was observed in all countries. Between 2008 and 2013, the annual number of travel-associated cases with heterosexual transmission fluctuated between 309 (2009) and 425 cases (2011). During the same period, the number of travel-associated cases with MSM transmission continuously increased from 53 in 2008 to 188 cases in 2013. Conversely, the number of travel-associated cases with unknown route of transmission continuously decreased from 84 in 2008 to 19 in 2013. Thus, the proportion of cases with MSM transmission among travel-associated cases with known transmission status increased from 14.1% in 2008 to 33.0% in 2013.

### Risk by country of residence, sex, age and destination

During 2008−2013, residents from Denmark, Finland, Norway and Sweden spent ca 1,320 million nights abroad (Table 2).

**Table 2:** Number of travel-associated cases of gonorrhoea, number of nights spent and risk by travel region, Denmark, Finland, Norway and Sweden, 2008−2013 (n = 3,224)

	Travel‑associated cases		Tourism nights (estimate 2008–2013)	Risk (cases/million nights)
**Region**	**Number**	**%**
**Total (all regions)**	**3,224**	**100**	**1,319,713,232**	**2.4**
Africa	169	5.2	52,777,817	3.2
The Americas				
North America	120	3.7	94,163,334	1.3
Central and South America	116	3.6	38,425,724	3.0
Asia	1,553	48.2	165,075,367	9.4
Europe				
EU 27	1,044	32.4	788,962,938	1.3
Other European countries	196	6.1	155,107,899	1.3
Oceania	26	0.8	25,200,155	1.0

EU: European Union.

While the number of travel associated cases are aggregated for the period 2008–2013, tourism nights were not available for all country-region combinations for all years. The denominator for the risk calculation is therefore the mean of tourism nights for the available years multiplied by six.

The most visited regions were the EU (59.8% of all nights spent), Asia (12.5%) and North America (7.1%). The overall risk for travel-associated gonorrhoea was 2.4 cases per million nights spent in outbound destinations (Table 2). The highest risk was found in Swedish travellers (3.1 cases per million nights) followed by Norwegian, Finnish and Danish travellers with 2.5, 2.2, and 1.2 cases per million nights, respectively. In Denmark, where tourism data were available by sex and age group, a higher risk for travel-associated gonorrhoea was observed in males compared with females (1.9 vs 0.3 cases per million nights, respectively) and among persons aged below 45 years, peaking at 2.2 cases per million nights for people aged 25–34 years (Table 3).

**Table 3:** Number of travel-associated cases of gonorrhoea, number of nights spent and risk by sex and age, Denmark, 2008−2013 (n = 365)

	Travel-associated cases		Tourism nights	Risk (cases/million nights)
Characteristic	Number	%	Number (mean 2012−2013)	
**Total**	**365**	**100**	**53,427,522**	**1.1**
Sex				
Male	315	86.3	27,881,556	1.9
Female	50	13.7	25,545,965	0.3
Age group (years)				
< 15	0	NA	NA	NA
15–24	86	23.6	8,659,638	1.7
25–34	116	31.8	8,929,735	2.2
35–44	84	23.0	8,653,369	1.6
45–54	50	13.7	11,391,493	0.7
55–64	20	5.5	8,526,496	0.4
≥ 65	9	2.5	7,266,792	0.2

NA: not applicable.

While the number of travel associated cases are aggregated for the period 2008–2013, tourism nights were only available for 2012−2013 by age and sex. The denominator for the risk calculation is therefore the mean of tourism nights for the available years multiplied by six.

Of the 3,224 travel-associated cases, 2,793 (86.6%) had acquired their infection either in Asia or Europe (Table 3). From December to July, Asia was the top destination for travel-associated gonorrhoea (53.4% of all travel-associated cases) peaking in January when Asia accounted for 60.6% of all travel-associated cases. From August to November, the top destination was Europe (48.6%) although the proportion of cases who had travelled to Asia remained substantial (36.5%). Monthly distributions of cases associated with travel to Africa, the Americas or Oceania did not show any obvious seasonal pattern. The highest risk was associated with travel to Asia with 9.4 cases per million nights. Travel to Africa and Central and South America was associated with a risk of ca 3 cases per million nights. Almost a third of all travel-associated cases were associated with a stay in Thailand, and the three destinations with the highest numbers of cases (Thailand, the Philippines and Spain) accounted for nearly half of all cases (Table 4).

**Table 4:** Top destinations for travel-associated gonorrhoea cases, Denmark, Finland, Norway and Sweden, 2008−2013

		Travel-associated cases	
**Rank**	**Destination country**	**Number**	**%**
1	Thailand	1,006	31.2
2	Philippines	258	8.0
3	Spain	229	7.1
4	Germany	199	6.2
5	Denmark	95	2.9
6	United Kingdom	93	2.9
7	United States	71	2.2
8	Turkey	67	2.1
9	Russia	65	2.0
10	Morocco	63	2.0
11	China	56	1.7
12	Sweden	51	1.6
13	France	50	1.6
14	Greece	47	1.5
15	Greenland	45	1.4
16	Indonesia	45	1.4
17	Brazil	42	1.3
18	Poland	37	1.1
19	Italy	29	0.9
20	Pakistan	27	0.8
	Other	649	20.1
	**Total**	**3,224**	**100**

Most cases with a probable country of infection in south-east Asia were reported with heterosexual transmission (98.3% for the Philippines and 95.9% for Thailand). Conversely, higher proportions of cases with MSM transmission were reported for top European destinations: in decreasing order, Germany (63.8%), the United Kingdom (59.1%), Spain (55.4%) and Denmark (55.2%). A comparable proportion of cases with MSM transmission was observed in people who travelled to the United States (55.9%). Top destinations for travel-associated gonorrhoea were very similar across all four Nordic countries with the exception of Greenland and Russia, which accounted for 12.3% and 9.0% of infections among Danish and Finnish travellers, respectively (Table 5).

**Table 5:** Top destinations for travel-associated gonorrhoea cases by country of residence, Denmark, Finland, Norway and Sweden, 2008−2013

Rank	Denmark			Finland			Norway			Sweden		
	Destination country	Travel‑associated cases		Destination country	Travel‑associated cases		Destination country	Travel‑associated cases		Destination country	Travel‑associated cases	
		Number	%		Number	%		Number	%		Number	%
1	Thailand	91	24.9	Thailand	217	40.9	Thailand	229	29.0	Thailand	469	30.5
2	Greenland	45	12.3	Russia	48	9.0	Philippines	77	9.7	Spain	119	7.7
3	Philippines	35	9.6	Philippines	40	7.5	Spain	75	9.5	Philippines	106	6.9
4	Germany	14	3.8	Spain	24	4.5	Germany	62	7.8	Germany	102	6.6
5	Spain	11	3.0	Germany	21	4.0	Sweden	31	3.9	Denmark	82	5.3
	Other	169	46.3	Other	181	34.1	Other	316	40.0	Other	660	42.9
	**Total**	**365**	**100**	**Total**	**531**	**100**	**Total**	**790**	**100**	**Total**	**1,538**	**100**

The proportion of travel-associated cases with a probable infection in Thailand decreased from ca 40% in 2008−2009 to 26.0% in 2013. Of the 376 foreign-born travel-associated cases, 154 (41.0%) travelled to their country of birth.

### Multivariable analysis

When adjusting for potential confounders, males were nearly three times more likely to have acquired their infection abroad compared with females (OR: 2.96, 95% CI: 2.62–3.34) (Table 6).

**Table 6:** Adjusted predictors of travel-associated gonorrhoea cases, Denmark, Finland, Norway and Sweden, 2008−2013 (n = 12,643)

Risk factor	OR	95% CI	p value	Cases exposed (%)
Sex				
Female	1 (Ref)	NA	NA	23.8
Male	2.96	2.62–3.34	< 0.01	76.2
Age group (years)				
< 25	1 (Ref)	NA	NA	35.3
25–34	1.40	1.24–1.57	< 0.01	32.8
35–44	2.38	2.09–2.71	< 0.01	18.1
45–54	3.51	3.01–4.10	< 0.01	9.3
55–64	4.36	3.47–5.49	< 0.01	3.4
≥ 65	6.04	4.12–8.84	< 0.01	1.1
Transmission				
MSM	1 (Ref)	NA	NA	32.8
Heterosexual	4.08	3.66–4.56	< 0.01	56.5
Other	2.15	1.03–4.47	0.04	0.4
Unknown	1.85	1.50–2.28	< 0.01	10.3
Country of residence				
Sweden	1 (Ref)	NA	NA	42.3
Denmark	0.22	0.18–0.26	< 0.01	27.2
Finland	1.37	1.15–1.62	< 0.01	12.3
Norway	1.48	1.26–1.73	< 0.01	18.2
Origin				
Native	1 (Ref)	NA	NA	38.1
Foreign-born	8.34	6.46–10.76	< 0.01	3.0
Unknown	1.28	1.10–1.49	< 0.01	58.9
Year				
2008	1 (Ref)	NA	NA	12.9
2009	1.04	0.88–1.23	0.65	13.3
2010	1.09	0.93–1.29	0.27	15.8
2011	0.98	0.83–1.15	0.81	16.7
2012	0.81	0.69–0.95	0.01	20.0
2013	0.83	0.71–0.98	0.03	21.4

CI: confidence interval; MSM: men who have sex with men; NA: not applicable; OR: odds ratio; Ref: reference value.

The risk of travel-associated gonorrhoea increased with age. Heterosexual transmission of gonorrhoea was four times more likely to be travel-associated compared with MSM (OR: 4.08, 95% CI: 3.66–4.56). Compared with natives, foreign-born cases were eight times more likely to be travel-associated (OR: 8.34, 95% CI: 6.46–10.76). With 2008 as reference, infections acquired in 2012 and 2013 were less likely to be travel-associated.

## Discussion

### Principal findings

Surveillance data from four Nordic countries suggested that at least 25% of gonorrhoea infections were related to travel, a proportion that slightly decreased over the study period. Half of these cases had a travel history in Asia, a continent that accounted for fewer than 15% of the nights spent abroad by the residents of these countries. Most of the travel-associated cases were observed among persons below 35 years of age. The Danish cases for which denominator information was available suggested that the highest risk for travel-associated gonorrhoea was among the 25–34 year-olds. Danish data also showed that the risk for travel-associated gonorrhoea in men is six times higher compared with women. This is consistent with the finding that younger people and men are more likely to report having a new sex partner while travelling [16]; younger people are also more likely to have a higher number of sex partners in general [17], which is a risk factor for having a new sex partner while overseas [16].

Although the reported number of cases of gonorrhoea decreased with age, the proportion of cases of gonorrhoea which were travel-related increased with age, with a peak of 59% among those aged 65 years or over; almost all of these cases were reported among heterosexual men. Very few cases of travel-related gonorrhoea were diagnosed among people aged 65 years and above and therefore older persons are at lower risk of acquiring gonorrhoea per million bed nights.

During the study period, the number of gonorrhoea and travel-associated gonorrhoea cases increased in all four countries, but the proportion of travel-associated cases decreased most recently in 2012−2013. Overall, this rise in reported cases has been mainly due to increasing numbers of cases among MSM, regardless of travel status [3], which is partly linked to continuing high-risk behaviour in this subpopulation [18], partly to increased testing and use of more sensitive tests such nucleic acid amplification tests [19]. The larger increase among locally acquired cases compared with travel-associated cases is likely to reflect the larger proportion of MSM among locally acquired cases. The increasing number of travel-associated cases with MSM transmission over the study period cannot be explained solely by improved data completeness for transmission status, but should be considered in the context of increasing reports of cases among MSM overall during this time period [3].

A disproportionate number of cases were associated with travel to Thailand and the Philippines. Although some of these cases are likely to be acquired through contact with sex workers, many travellers, particularly backpackers, often find sexual partners among other travellers. The risk of new partner acquisition and overall risk of unsafe sex among backpackers in Thailand has been found to be associated with male gender and longer trip length [20]. Backpackers are also less likely to use condoms when having sex with travel partners compared than with commercial sex workers [21]. Thailand is reported to be the foreign country where the largest number of Swedish males contract HIV and many Swedish men have sex with commercial sex workers while on holiday there, leading to risk of transmission of STI also from the local population [22].

South-east Asian countries are reported to have high levels of multidrug-resistant *N. gonorrhoeae* [23]. Information on the laboratory test used to confirm gonorrhoea is not available within the European surveillance system. This makes it difficult to know how often culture and subsequent susceptibility testing were performed among travel-associated cases. Considering the current concerns on antimicrobial-resistant gonorrhoea in Europe and globally [24], monitoring susceptibility of strains acquired during travel is important.

Over half of gonorrhoea infections acquired in the top European destinations were in MSM. This reflects the high prevalence of MSM having sex abroad, reported to be 25% in the previous 12 months from the European Men-Who-Have-Sex-With-Men Internet Survey (EMIS), and the low proportion of condom use during anal intercourse while abroad [25]. The top destinations for sex abroad reported in EMIS were also Spain and Germany, as in our study. Barcelona and Berlin are both extremely popular sex tourism hotspots in Europe for MSM.

Foreign-born cases were more likely to be travel-associated compared with natives. Approximately 40% of travel-associated foreign-born cases travelled to their country of birth, probably visiting friends or relatives (VFR). VFR travellers are a well-identified risk group for travel-associated illness although the definition may mask a more complex reality [26].

### Strengths and weaknesses of the study

The study is based on a relatively homogeneous population in the Nordic European countries. A substantial proportion of Nordic residents travel abroad and this makes the Nordic population very suitable for estimating risk of travel-associated conditions. The surveillance systems for STIs in these countries have been stable during this time, with good case ascertainment and availability of diagnostics. Unfortunately it was not possible to incorporate analysis of Euro-GASP data due to the low completeness of the relevant variables. Improving the completeness of these variables in Euro-GASP should be a priority to allow for better monitoring of the resistance patterns of strains imported into Europe.

The travel behaviour across the included countries is also rather similar: residents of Nordic countries tend to travel to warmer countries during the darkest and coldest months of the year. Travel tourism data were collected mostly via household survey, a method which is prone to memory bias. However, the number of nights spent by destination was fairly stable over the study period suggesting that these data were reliable. Our decision to average the number of nights spent for the study period may have masked annual variations, especially for destinations with fewer nights spent, such as Africa or Oceania. For such destinations the estimated risk may be less accurate.

Unfortunately, tourism data were not available by age group and sex for Finland, Norway and Sweden, limiting enhanced analysis of the data. In Denmark where tourism data were available by age group, there was little variation over 2012−2013. Information on the purpose of travel was not available for cases. Therefore, we were not able to differentiate leisure tourism from business travel or family visits.

### Comparison with other studies

The data presented in this paper are consistent with data reported by Steffen et al. who estimated a risk of travel-associated gonorrhoea of 0.06% per month of stay in developing countries [27]. Our findings indicate the highest risk was associated with travel to Asia with 9.4 cases/million nights, which would correspond to an incidence rate of 0.03% per month.

The high proportion of travel-associated cases in older age groups has previously been reported in Sweden where more than 50% of gonorrhoea cases aged 35 years or over were infected abroad during 2007−2011, compared with 20% to 25% for persons below 35 years of age [28]. The Swedish study found that women and MSM were more frequently infected in Sweden than heterosexually infected men. A recent systematic review of casual sex and foreign travel found that people engaging in casual sex while abroad are more likely to be young and males [29].

### Possible explanations and implications for clinicians and policymakers

The results presented here highlight the role of young adults below 35 years of age in the epidemiology of gonorrhoea, including travel-associated gonorrhoea. International guidelines recommend specifically addressing STI during pre-travel consultations [7] but a recent systematic review underlined the low level of evidence on the impact of standard STI pre-travel advice on sexual behaviour [30]. The same review also found that motivational pre-travel STI interventions (described as ‘a directive, client-centred counselling style for eliciting behaviour change by helping clients to explore and resolve ambivalence’ [31]) were not superior to standard STI travel advice [30]. One cohort study identified in this review, however, did find that recall of reading STI information appeared to be related to more consistent condom use [30]. Since a large proportion of travellers do not seek advice in pre-travel consultations [32], other channels of information such as social media including dating apps could be explored. Young adults could be targeted with safe-sex messages related to travel when visiting STI or youth clinics: persons with a history of multiple sex partners and/or an STI are at risk of travel-associated casual sex [29]. The role of social media in targeting prevention messages needs to be considered and further investigated: research suggests that social media plays an increasing role as information sources for travellers [33,34]. Apart from social media, online resources are extensively used by all age groups for travel planning [34]. Targeted online prevention messages could be considered at peak travel periods together with research on their effectiveness.

Apart from the safe-sex message, young adults should be educated to know that a large proportion of gonorrhoea cases are asymptomatic and hence, after risky behaviour, testing is important irrespective of symptoms.

Finally, these data indicate that older males have a higher likelihood of having been infected abroad when presenting with a gonorrhoea infection. This should be taken in consideration by clinicians when treating these patients. Although they represent a lesser burden when compared with younger age groups, this population should not be forgotten by public health interventions.
